# Salivary Cortisol Concentration Is an Objective Measure of the Physiological Response to Loud Music

**DOI:** 10.3390/audiolres14060090

**Published:** 2024-12-09

**Authors:** Robert Tomljenović, Andro Košec, Livije Kalogjera, Ivana Ćelap, Domagoj Marijančević, Davor Vagić

**Affiliations:** 1 Department of Otorhinolaryngology and Head and Neck Surgery, University Hospital Center Sestre Milosrdnice, Vinogradska Cesta 29, 10000 Zagreb, Croatia; robert.tomljenovi@gmail.com (R.T.); kalogjera@sfzg.hr (L.K.); davor.vagic@kbcsm.hr (D.V.); 2 School of Medicine, University of Zagreb, Šalata 2, 10000 Zagreb, Croatia; 3 Department of Clinical Chemistry, Sestre Milosrdnice University Hospital Center, 10000 Zagreb, Croatia; ivana.celap@gmail.com (I.Ć.); domagoj.marijancevic@kbcsm.hr (D.M.); 4 School of Dental Medicine, University of Zagreb, Gundulićeva 5, 10000 Zagreb, Croatia

**Keywords:** physical stress, psychological stress, stress response, salivary cortisol, loud music, noise

## Abstract

**Purpose**: This study examines the potential associations between salivary cortisol concentrations and subjective stress test scores in healthy individuals subjected to sound-related, psychological, and physical stressors. **Methods**: This study employed a single-center observational cross-sectional design, with a sample size of 36 subjects recruited from a tertiary referral audiology center. Between 2023 and 2024, the study recruited subjects with normal hearing, baseline salivary cortisol levels, and subjective stress levels. The participants were requested to complete an STAI-Y1 questionnaire and provide salivary cortisol samples before and following exposure to sound-related, psychological, and physical stress tests. **Results**: Exposure to psychological and physical stressors significantly increased STAI-Y1 scores (Friedman’s test, χ^2^ = 57.118, df = 2, *p* = 0.377). This increase was greater than that observed in response to loud, favorite music (Friedman’s test, χ^2^ = 57.118, df = 2, *p* < 0.0001). The salivary cortisol concentration significantly increased in all three provocation tests (Friedman’s test, χ^2^ = 95.264, df = 5, *p* < 0.0001). Furthermore, there is no significant difference in salivary cortisol concentrations between the three pre-test and post-test measurement intervals, indicating a comparable stress-inducing pattern regardless of the nature of the stimulus (Friedman’s test, χ^2^ = 95.264, df = 5, *p* > 0.05). **Conclusions**: Exposure to loud favorite music increases salivary cortisol concentrations, as does acute physical and psychological stress. Interestingly, unlike psychological and physical stress, loud music was not objectively perceived as stress, which may mask the physiological signs of stress, potentially increasing the risk of both acute and chronic stress-related health outcomes.

## 1. Introduction

Sound is an integral part of our daily environment. Typically, the sounds we encounter fall below levels that can harm our hearing. However, noise-induced hearing loss (NIHL) can occur from a single exposure to an intense sound, such as an explosion, or from prolonged exposure to loud noises, such as those experienced in nightclubs [1]. Continuous exposure to such environments can lead to the release of cortisol, a stress hormone, which may adversely affect cardiovascular health [2].

Cortisol, the primary glucocorticoid hormone, is a crucial indicator of the body’s stress response. It is the main product of the neuroendocrine system’s hypothalamic–pituitary–adrenal (HPA) axis, activated when an individual perceives stress. During stress, the HPA axis triggers a cascade of physiological events, resulting in elevated cortisol concentration. Prolonged cortisol secretion signals the body that it is under constant threat, potentially leading to disorders such as hypertension, thyroid dysfunction, heart disease, diabetes, obesity, and depression.

Under normal conditions, cortisol follows a circadian rhythm, peaking in the morning and gradually decreasing throughout the day without stressors [3]. Typical morning cortisol concentrations range from 15.5 ± 0.8 nmol/L, while nighttime levels drop to 3.9 ± 0.2 nmol/L. The disruption of this rhythm, primarily through prolonged exposure to environmental stressors like noise, can have significant health implications [4].

According to the World Health Organization (WHO), traffic-related noise contributes to over 1.5 million years of healthy life lost annually in Western Europe alone, with ischemic heart disease accounting for 61,000 of these years [5].

While often considered soothing, music can also affect cardiovascular and emotional responses, although the exact mechanisms behind these effects remain unclear [6]. Some research suggests that music may reduce the sympathetic nervous system and neuroendocrine activity, serving as a potential method of stress relief [7].

Loud noise has been shown to influence the HPA axis, leading to elevated cortisol concentration in both serum and saliva. Chronic noise exposure may independently contribute to cerebrovascular risk by affecting hemodynamics, hemostasis, and oxidative stress, potentially leading to cardiovascular disease [8]. The combination of noise and psychological stress appears to synergistically elevate cortisol concentration, further amplifying the risk of health issues [9]. Studies indicate that fast-tempo music may increase sympathetic nerve activity while slower music can lower heart rate and induce relaxation [10,11]. However, a major concern arises from the fact that individuals listening to loud yet enjoyable music may not recognize the stress they are under, putting them at greater risk for long-term cardiovascular consequences. Research by Sing et al. has demonstrated that sound levels above 92 dBA stimulate the sympathetic neuroendocrine system, releasing adrenaline and noradrenaline, while sounds over 120 dBA trigger cortisol release in humans and animal models [12]. Moreover, continuous exposure to sounds exceeding 85 dB for eight hours has been linked to NIHL [13].

The research hypothesis is that exposure to loud and pleasant music increases salivary cortisol concentration, similar to acute psychological and physical stress. Understanding the connection between loud music exposure and salivary cortisol concentration in healthy individuals could provide valuable insights into the relationship between stress markers and hearing impairment. Additionally, exploring the association between cortisol fluctuations and acute stress—whether psychological, physical, or noise-induced—may enhance our ability to assess hearing damage risk and elucidate the interactions among these factors.

## 2. Materials and Methods

This is a single-center, cross-sectional observational study on healthy human subjects in a tertiary audiology center. The samples and tests were obtained during 2023 and 2024 and assembled according to STROBE guidelines.

The inclusion criteria required subjects to be between 20 and 30 years old (Table 1) and have no pre-existing conditions affecting hearing, balance, or serum/salivary cortisol concentration.

Subjects needed to complete all testing phases and provide full documentation and questionnaires. Before study inclusion, all subjects underwent pure tone average (PTA), and only those with normacusis—defined as a PTA < 25 dB at the speech-discriminating frequencies (500 Hz, 1000 Hz, 2000 Hz, and 4000 Hz)—were enrolled (Table 2). This analysis did not include additional audiological tests, such as tympanometry, acoustic reflex, and otoacoustic emissions. Exclusion criteria included a history of psychological or mental illness, particularly those affecting chronic stress levels (e.g., anxiety or clinical depression). Ultimately, 36 healthy participants were included in the study, 18 male and 18 female.

The study protocol was approved by the Ethics Committee of University Hospital Sestre milosrdnice, Zagreb School of Medicine, and School of Dentistry. Written informed consent was obtained from all participants.

Participants were asked to assess their general anxiety and stress levels using the validated State-Trait Anxiety Inventory (STAI) questionnaire. Salivary cortisol samples were analyzed in the biochemical laboratory at University Hospital Center Sestre milosrdnice.

Cortisol concentration was determined using Elecsys Cortisol II assay (catalog number: 07027150190) on the automated Cobas e 801 analyzer (Roche Diagnostics GmbH, Mannheim, Germany) using an electrochemiluminescent immunoassay based on a competitive principle. In the first incubation step, competition occurs between the cortisol analyte in the saliva sample and the analyte derivative labeled with a ruthenium complex, in competition with an antibody labeled with biotin. During the second incubation, the complex binds to paramagnetic particles coated with streptavidin via biotin. Upon the application of a voltage to the electrode surface, a chemiluminescent reaction takes place between the ruthenium complex and tripropylamine. Photon emission is inversely proportional to the concentration of cortisol analyte in the saliva sample. The method is standardized according to the IRMM/IFCC-451 standard using the reference method ID-GC/MS (Isotope Dilution-Gas Chromatography/Mass Spectrometry). The Elecsys Cortisol II assay has a declared measuring range of 3.0–1750 nmol/L, a limit of quantification of 3.0 nmol/L, an intermediate precision (CV) of 4.9% (at 9.79 nmol/L), and 3.4% (at 28.5 nmol/L). Samples were collected in Salivette containers (Sarstedt, Germany) and immediately stored at 2–8 °C. A minimum volume of 2 mL of saliva, collected passively, was required for adequate analysis. Before collection, participants rinsed their oral cavities with drinking water to prevent contamination.

STAI is the “gold standard” for measuring acute stress [14]. It comprises separate self-report scales for measuring two distinct anxiety concepts: state anxiety and trait anxiety. The reliability and validity of the STAI are well reported (Cronbach’s alpha = 0.896). The STAI-Y2 scale consists of 20 statements that ask people to describe how they generally feel. The STAI-Y1 scale also consists of 20 statements, but the instructions require subjects to indicate how they feel at a particular moment. The STAI-Y1 scale can be used to determine the actual levels of anxiety intensity induced by stressful situations. Each question is answered on a four-point Likert scale (1: not at all, 2: a little, 3: moderately, and 4: a lot).

The results range between 20 and 80 points, and the subjects are then divided into three groups according to their stress and anxiety levels: 20–37 (not stressed), 38–44 (moderately stressed), and 45–80 (very stressed). A previously established cut-off value of 38 was taken as a disqualifying value, with all the participants passing through to have their salivary cortisol measured. We used part Y-1, which was translated into Croatian, and validated STAI with the purchased license [15].

### 2.1. Experimental Protocol

The study consisted of three tests designed to assess the impact of loud music and psychological and physical stress on salivary cortisol concentration.

1. Loud music exposure Test

All participants completed a baseline STAI-Y1 form at 8.50 pm to assess their stress levels without external stressors. Salivary cortisol samples were collected at the same time. Participants were instructed to refrain from smoking, consuming alcohol, or using cosmetics two hours before sampling to avoid contamination. Afterward, participants attended a one-hour music concert by a performer they liked to minimize bias due to individual musical preferences. The sound level was 90 dB, with a maximum of 95 dB, measured using a Benetech GM1351 noise sensor. After the concert, at 10 pm, salivary cortisol and STAI-Y1 scores were reassessed. A follow-up PTA was performed the next day to ensure no transient threshold shifts due to acoustic trauma.

2. Trier Social Stress Test (TSST)

Following the baseline measurement of cortisol and STAI-Y1 the day after the concert, participants underwent the Trier Social Stress Test (TSST) at 9 pm. This validated test induces psychological stress in a controlled environment, consisting of three five-minute phases. In the first phase, participants prepared a presentation on an unfamiliar topic before three examiners and a video camera. In the second phase, they delivered the presentation without notes. Finally, participants performed serial subtraction by subtracting 13 from 1022 as quickly as possible, restarting after any errors. After the TSST, salivary cortisol and STAI-Y1 scores were collected again.

3. Cold Pressor Task (CPT)

Participants underwent a cold pressor task at 9 pm the day after the Trier Social Stress Test. The final test involved inducing physical stress using the cold pressor task (CPT), where participants submerged a hand or forearm in 0 °C water for 120 s. This gradually induces mild to moderate pain, with cortisol concentration peaking approximately 15 min post-task. Before and after this test, participants completed the STAI-Y1 test, and salivary cortisol was measured. All participants completed the task without removing their limbs before the minimum time elapsed.

### 2.2. Statistical Analysis

A sample power analysis was made based on the published cut-off values of the STAI-Y1 questionnaire and known reference values of salivary cortisol. Alpha was set at 0.05, and the power of the test was 80%. Power analysis set the minimum required sample size to establish statistical significance at 31 subjects in the patient group.

We used STAI-Y1 scores and salivary cortisol values as continuous variables and gender and stress class as categorical variables. The data distribution was calculated using the Kolmogorov–Smirnov test. Statistical analysis was performed depending on the normality of the distribution using the Mann-Whitney U test, and post-hoc analysis with the Friedman test for paired samples was then performed to assess correlations (the non-parametric alternative to the one-way ANOVA with repeated measures) between salivary cortisol and STAI-Y1 values. ROC curve testing was performed to identify salivary cortisol cut-off values, with the subjects’ highest specificity and sensitivity associated with high-stress responses. Spearman correlation analysis was performed between the individual values of salivary cortisol concentration and the scores of psychometric instruments. All tests of statistical significance were performed using a two-sided 5% type I error rate.

Statistical analysis was performed using the MedCalc software (Version 11.2.1^©^ 1993–2010. MedCalc Software bvba Software, Broekstraat 52, 9030 Mariakerke, Belgium) and SPSS (Version 22.0., 2013. IBM SPSS Statistics for Windows, Armonk, NY, USA: IBM Corp.) using standard descriptive statistics and frequency tabulation as indicated.

## 3. Results

**Figure 1:**
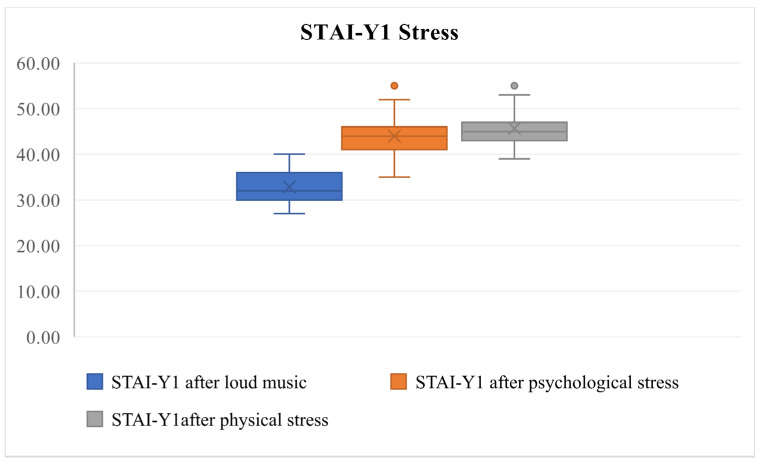
Comparison of STAI-Y1 stress values across conditions following the loud music, psychological, and physical discomfort tests using Friedman’s test (Friedman’s test, χ^2^ = 57.118, df = 2, *p* < 0.0001). Outliers (dots) indicate values significantly deviating from the interquartile range, reflecting individual variability.

Thirty-six participants (18 males and 18 females) completed the study, with a mean age of 26.7 years (Figure 1). All participants had regular PTA (<25 dB) (Table 2) and scored below 38 points on the pre-test STAI-Y1 questionnaire, indicating no significant baseline stress levels before testing.

The Mann–Whitney U test and Spearman correlation analysis were used to see whether age or sex is associated with cortisol concentration or stress scores. There were no significant correlations or differences between the variables.

Three subjective stress classes were identified, with 35/36 participants in the no-stress cluster and 1/32 in the moderate-stress class after 1 h of listening to favorite music at 90–95 dB SPL. After exposure to psychological stress, 2/32 subjects scored their stress levels as no-stress, 16/32 as moderate stress, and 18 in the high-stress class. After exposure to physical discomfort, no subjects scored as no-stress, 13/32 identified their stress levels as moderate, and 23 scored in the high-stress class.

Friedman’s test was used to compare repeated measures of stress levels (STAI-Y1 scores) across the three conditions: loud music, psychological discomfort, and physical discomfort (Friedman’s test, χ^2^ = 57.118, df = 2, *p* < 0.0001, Figure 1). The test revealed that psychological and physical discomfort significantly increased stress levels compared to loud music. At the same time, no significant difference was found between stress levels induced by psychological and physical discomfort (Friedman’s test, χ^2^ = 57.118, df = 2, *p* = 0.377).

**Figure 2:**
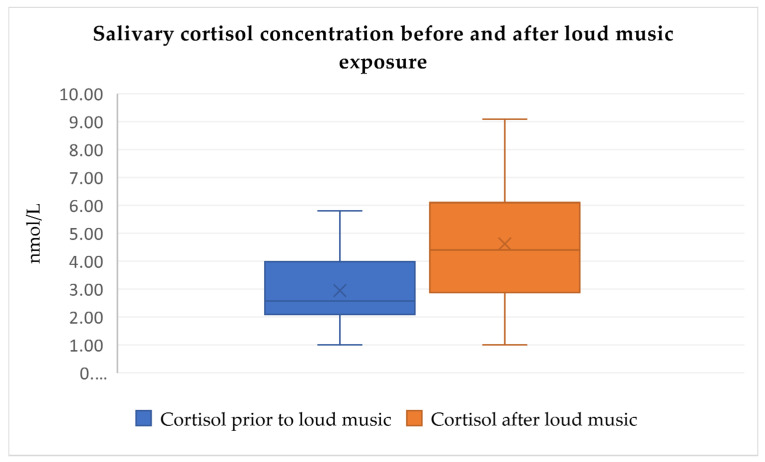
Salivary cortisol concentration increases significantly after loud music exposure test (medians and IQR, Friedman’s test, χ^2^ = 95.264, df = 5, *p* < 0.0001).

**Figure 3:**
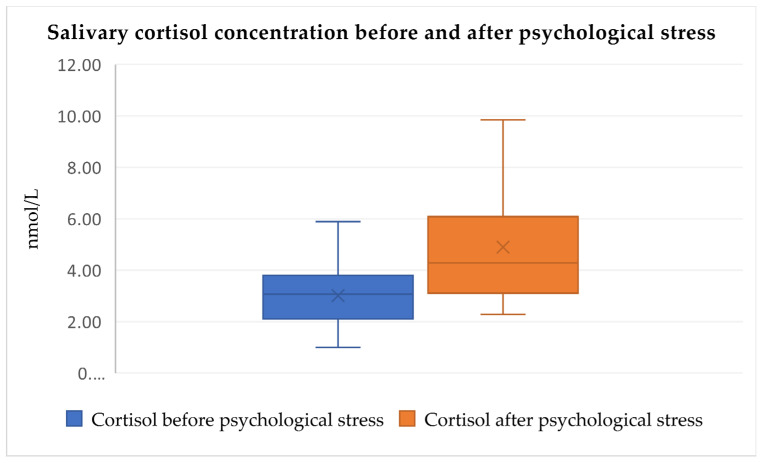
Salivary cortisol concentration increases significantly after psychological stress test (medians and IQR, Friedman’s test, χ^2^ = 95.264, df = 5, *p* < 0.0001).

**Figure 4:**
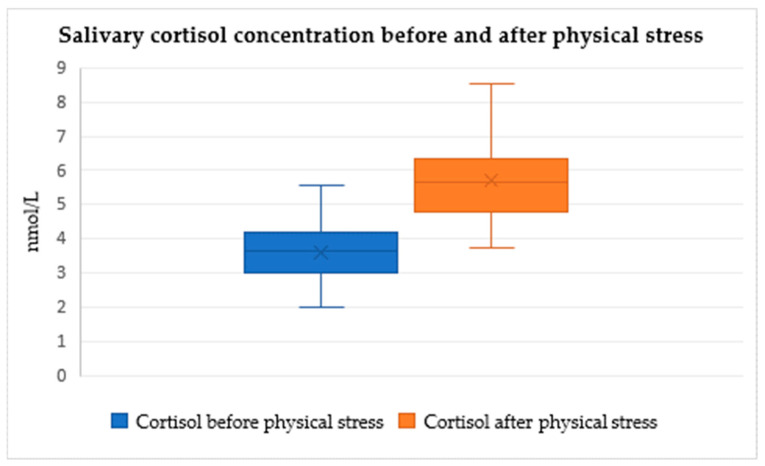
Changes in median values of salivary cortisol concentration before and after physical discomfort tests (CPT) (medians and IQR, Friedman’s test, χ^2^ = 95.264, df = 5, *p* < 0.0001).

When analyzing the changes in salivary cortisol concentration before and after the three tests, Friedman’s test showed that salivary cortisol concentration rises significantly in all three paired provocation tests when comparing post-test and pre-test values (Friedman’s test, χ^2^ = 95.264, df = 5, *p* < 0.0001, Figure 2, Figure 3 and Figure 4). No significant differences were observed between pre-test cortisol levels among the three conditions, suggesting comparable baseline stress levels. Post-test cortisol concentrations did not differ significantly between the three stressors, indicating a consistent physiological response regardless of stimulus type. Outliers, represented by individual dots in the box plots, indicate variability in participants’ cortisol response (Friedman’s test, χ^2^ = 95.264, df = 5, *p* > 0.05).

The median values and interquartile ranges (IQR) for salivary cortisol concentrations across the different tests are summarized in Table 3.

**Figure 5:**
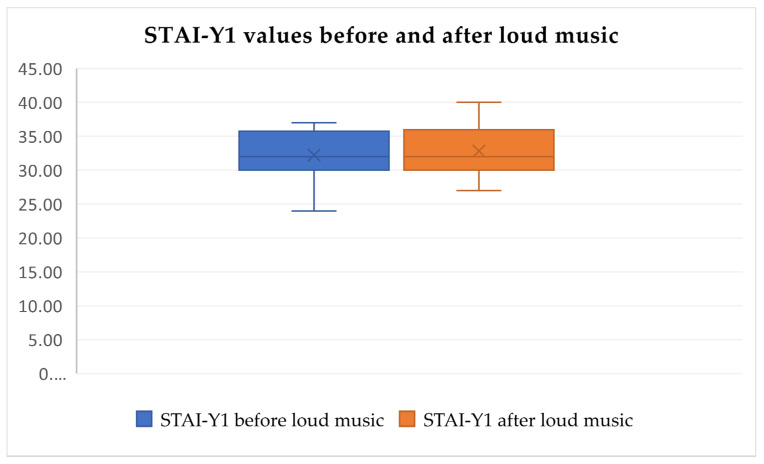
STAI-Y1 scores do not change significantly after loud music exposure (Friedman’s test, χ^2^ = 133.892, df = 5, *p* > 0.05).

**Figure 6:**
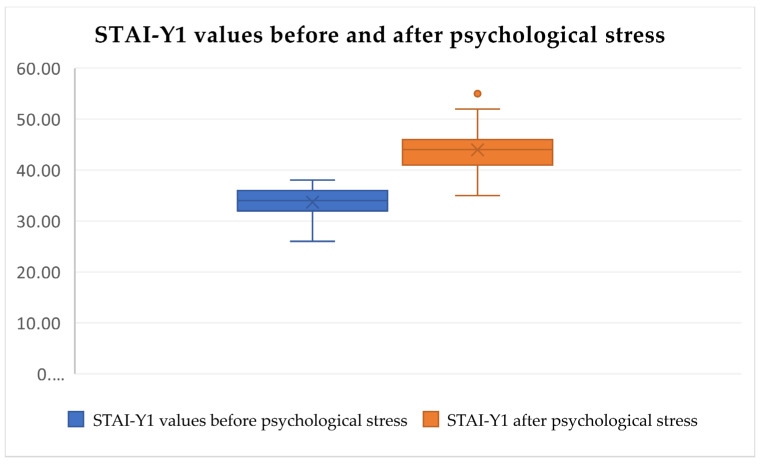
STAI-Y1 scores increase significantly after psychological discomfort tests. The dots represent outliers with notably higher stress scores (Friedman’s test, χ^2^ = 133.892, df = 5, *p* < 0.0001).

**Figure 7:**
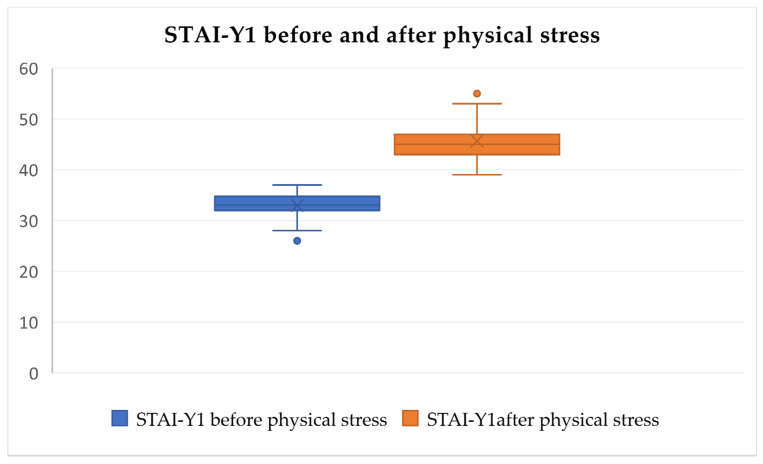
STAI-Y1 scores significantly increase after physical discomfort tests. The dots represent outliers with notably higher or lower stress scores (Friedman’s test, χ^2^ = 133.892, df = 5, *p* < 0.0001).

After analyzing the pre-test and post-test STAI-Y1 values, statistically significant differences in increased psychological and physical discomfort stress levels were identified after the tests (Friedman’s test, χ^2^ = 133.892, df = 5, *p* < 0.0001, Figure 5, Figure 6 and Figure 7) but were not identified in the pre-test and post-test values related to loud music exposure (Friedman’s test, χ^2^ = 133.892, df = 5, *p* > 0.05, Figure 5). In addition, the subjects subjectively rated the physical discomfort test as being equally stressful as the psychological stress test (Friedman’s test, χ^2^ = 133.892, df = 5, *p* > 0.05). Both were significantly more stressful than loud music exposure (Friedman’s test, χ^2^ = 133.892, df = 5, *p* < 0.0001).

To investigate whether pre-test and post-test salivary cortisol concentration could predict a severe stress response and reduce test–retest variability, we reassessed the STAI-Y1 stress scores. Patients were grouped into either a combined low and intermediate stress group or a severe stress group based on an STAI-Y1 cut-off value of 45. We then performed ROC curve analysis to identify the pre-test and post-test salivary cortisol cut-off values with the highest sensitivity and specificity for predicting severe stress after physical and psychological stress tests. We used the Youden J index for dichotomous outcomes.

The analysis showed that a pre-test salivary cortisol cut-off of ≤2.35 for physical stress was associated with a severe stress response (AUC = 0.533, *p* = 0.7465, sensitivity = 21.74%, specificity = 92.31%). For psychological stress, a pre-test cut-off of ≤3.36 was found to be associated with a severe stress response (AUC = 0.546, *p* = 0.648, sensitivity = 72.22%, specificity = 55.56%).

Loud music exposure was not included in this analysis as the STAI-Y1 scores did not change before and after exposure, suggesting it did not affect the stress response in this study.

Spearman correlation analysis showed that between the individual values of salivary cortisol concentration and the scores of psychometric instruments, there is a significant correlation between STAI-Y values after music exposure and salivary cortisol concentration before music exposure (0.375, *p* = 0.024). In addition, there is a significant correlation between salivary cortisol concentrations after music exposure and physical stress (0.553, *p* = 0.001) but not between STAI-Y values, regardless of psychometric test type. No correlations were identified between salivary cortisol concentrations after psychological stress and psychometric test values.

## 4. Discussion

Longstanding indirect evidence shows that loud sounds, including concert music, are environmental stressors. Several studies have implied that continuous exposure to loud music results in measurable alterations in human homeostasis parameters.

This can be explained by the intricate interaction between the auditory and limbic systems, which are deeply involved in processing emotional and sensory stimuli. The amygdala, a central structure in the limbic system, is particularly sensitive to sounds that carry emotional significance or meaning, such as music, vocalizations, and even distressing sounds like crying. Its primary role in emotional processing involves the detection of these sound cues, which can trigger appropriate emotional responses. Specifically, the amygdala plays a critical role in auditory fear conditioning, wherein exposure to certain sounds becomes associated with fear, and it regulates the acoustic startle response—an automatic reaction to sudden or intense sounds. Additionally, the amygdala can modulate the plasticity of the auditory cortex, influencing how auditory signals are processed and interpreted over time. This is particularly relevant in the context of stress, as exposure to a stressful acoustic stimulus can activate the amygdala, inducing the release of stress hormones through the hypothalamic–pituitary–adrenal (HPA) axis. This physiological response is associated with the perception of discomfort and anxiety.

However, not all acoustic stimuli induce negative responses. For example, music experienced in a positive, contextually enriching environment, such as at a concert or therapeutic session, can produce a different neurophysiological response. In this context, the amygdala may respond less intensively, and the overall stress response may be reduced, reflecting a more pleasurable and engaging auditory experience. This suggests that the emotional valence associated with sound—negative or positive—plays a critical role in determining the subsequent physiological and emotional outcomes [16].

To date, there have been no reported data on direct comparisons between cortisol concentration, subjective anxiety, and stress response depending on exposure to loud music and physical and psychological stress in a single patient cohort. To address this gap, this study analyzed data supporting the hypothesis that loud music exposure, such as that from concerts, disrupts salivary cortisol regulation. Connections between exposure to loud music and changes in salivary cortisol concentration provide us with new information about the salivary stress marker link. Additionally, correlations between salivary cortisol and subjective stress levels offer insight into systemic stress response. Salivary cortisol measurements are straightforward and reliably reflect free cortisol concentration in the blood. They have been utilized in studies examining road traffic and aircraft noise exposure [17].

Our results indicate that exposure to psychological and physical discomfort significantly raises stress levels compared to loud music (Friedman’s test, χ^2^ = 57.118, df = 2, *p* < 0.0001, Figure 3). Importantly, no significant difference was found between stress levels associated with psychological and physical discomfort (Friedman’s test, χ^2^ = 57.118, df = 2, *p* = 0.377). Despite a wide range of physiological effects that influence cardiovascular and hormonal activity, our findings reveal that 35 out of 36 participants fell into the no-stress cluster, with only one out of 36 categorized as experiencing moderate stress after loud music exposure. This suggests that individuals typically do not perceive discomfort from listening to music.

It is possible that participants did not perceive themselves as under significant stress during exposure to loud music at a concert because such environments are often associated with positive experiences. Concerts are typically enjoyable and engaging, which may influence how individuals perceive stress. The context of the noise, whether it is pleasurable (e.g., music at a concert) or aversive (e.g., traffic or industrial noise), can affect stress perception and physiological responses. Positive experiences can lead to an effect where the body’s objective markers, such as cortisol concentration, rise due to loud music exposure. However, subjective stress is not reported because the experience is enjoyable.

It is critical to consider that the excitement and positive emotions associated with attending a concert could also influence cortisol concentration. While loud sound exposure is generally considered a stressor, the context in which it occurs—such as the enjoyable and stimulating environment of a concert—may modify the perception of stress and impact the body’s physiological response.

Conversely, when analyzing the relationships between salivary cortisol before and after the three tests, Friedman’s test showed that salivary cortisol values rise significantly in all three paired provocation tests when comparing post-test and pre-test values (Friedman’s test, χ^2^ = 95.264, df = 5, *p* < 0.0001, Figure 5, Figure 6 and Figure 7). Additionally, cortisol concentration did not significantly differ among the three measurement intervals, indicating a consistent stress-inducing pattern regardless of the stimulus (Friedman’s test, χ^2^ = 95.264, df = 5, *p* > 0.05). This supports the notion that sound acts as a non-specific stressor, activating both the autonomic nervous system and the endocrine pathway. Zumanian et al. [7] concluded that excessive sounds significantly affect cortisol concentration, corroborating our study’s findings [18].

When assessing the association between pre-test and post-test STAI-Y1 values, we identified statistically significant increases in psychological and physical discomfort stress levels after the tests. At the same time, no differences were noted for loud music exposure. Participants rated the physical discomfort test as equally stressful as the psychological stress test and significantly more stressful than loud music exposure. Chukwubuike et al. investigated the effect of music on preoperative anxiety in cataract surgery patients using the Hamilton State-Trait Anxiety Inventory (STAI). They found that music positively influenced preoperative anxiety, which aligns with our pre-test and post-test STAI-Y1 values. While their study lacked salivary cortisol measurements, our findings indicate a similar pattern in anxiety response.

Moderate exposure (e.g., exercise or testing) increases the secretion of stress hormones and results in favorable physiological adaptations. In contrast, stimuli causing mild, short stress episodes or prolonged exposure to high levels of detrimental stress may result in the under- or over-activation of physiological adaptation mechanisms [19].

We would also point out that our data seem to indicate a significant interaction between loud sounds and stress levels, which elegantly supports the Arndt–Schulz law, especially when translated into Selye’s syndrome—an expression of Claude Bernard’s “milieu intérieur” [20]. Selye’s general adaptation syndrome shows that continuous exposure to stress factors leads to exhaustion, which may have fatal consequences. A stress reaction is not a specific reaction but rather a general reaction. Selye’s general adaptation syndrome shows that continuous exposure to stress factors leads to exhaustion, which may have fatal consequences. The stress reaction is not a specific reaction but rather a general reaction.

These findings suggest that salivary cortisol concentration can provide some insight into stress responses. However, its predictive value for severe stress remains limited, particularly in distinguishing between different stress types. This highlights the need for further refinement and exploration of additional biomarkers.

### Study Limitations

However, some study limitations should be acknowledged. One significant limitation of this study is the narrow age range of participants (20–30 years old). This restricts the generalizability of the findings to older or younger populations. Individuals in different age groups may exhibit varying behaviors, perceptions, and physiological responses, so the study’s conclusions may not apply to a broader demographic. While the study investigated loud music and psychological and physical stress, other relevant stressors (e.g., social stress, environmental stress) were omitted, which may provide a more comprehensive understanding of stress responses. Another limitation of this study is the participants’ lack of detailed medical history. Combined with the potential impact of short-term withdrawal from substances like alcohol and nicotine—which may also influence cortisol regulation—these limitations highlight the need for careful participant screening and control in future research to better isolate the effects of the tested stressors. In addition to the previously mentioned limitations, it is essential to acknowledge that the study did not include a control experiment where participants listened to their favorite music at a lower sound pressure level. A control experiment, where participants listen to their favorite music at a lower volume, would help differentiate between the effects of music-induced emotional arousal and those directly linked to the stressors under investigation. This addition would enhance the ability to isolate the specific impact of the stressors tested in this study and further validate the conclusions drawn.

## 5. Conclusions

This study demonstrated that psychological and physical discomfort significantly increased stress levels compared to loud music, as measured by STAI-Y1 scores. Participants consistently reported higher stress levels following psychological and physical stress, with no significant difference between these two stressors; both were rated as more stressful than loud music exposure. Salivary cortisol concentration rose significantly in response to all three stressors, confirming the physiological impact of each. However, pre-test and post-test cortisol concentration did not vary significantly between stressors, indicating that the body’s physiological response to stress may be similar regardless of the type of stressor. Additionally, the predictive power of salivary cortisol concentration for identifying severe stress responses was limited, with low sensitivity and specificity observed in the ROC analyses. These findings suggest that while cortisol is a reliable marker for stress, its ability to differentiate between levels of stress severity is limited, and psychological and physical stressors provoke more robust subjective stress responses than loud music. Future research should explore additional biomarkers to improve the prediction of stress severity and consider interventions targeting the more potent stress effects of psychological and physical discomfort.

## Figures and Tables

**Table 1:** Mean Age, Standard Deviation, and Age Range by Gender.

Group	Mean Age (y)	SD Age (y)	Min-Max Age (y)
Total	27.61	1.09	26–29
Males	25.72	2.22	22–29
Females	26.67	1.97	22–29

**Table 2:** Audiometric Thresholds (dB) by Group: Mean, Standard Deviation, and Range.

Group	Mean Threshold (dB)	SD (dB)	Min–Max (dB)
Total	12.85	2.74	10.0–20.0
Males	13.08	2.64	10.0–20.0
Females	12.83	2.84	10.0–20.0

**Table 3:** Median and Interquartile range (IQR) values of salivary cortisol concentration, [nmol/L].

Test	Median	Interquartile Range (IQR)
Before noise exposure test	2.58	1.53
After noise exposure test	4.29	2–78
Before psychological stress test	3.26	1.49
After psychological stress test	5.76	4.11
Before physical stress test	3.65	1.18
After physical stress test	5.65	1.20

## Data Availability

Data are available under reasonable request to the corresponding author.
